# Triceps aponeurosis and deltoid tuberosity as a landmarks for radial nerve dissection: a cadaveric study

**DOI:** 10.1016/j.jseint.2024.03.017

**Published:** 2024-04-13

**Authors:** Feras Qawasmi, Lena Qawasmi, Hazem Safadi, Suhas P. Dasari, Mustafa Yassin

**Affiliations:** a Department of Orthopedic Surgery, Hasharon Hospital, Petah Tikva, Israel; b Jerusalem Shoulder and Elbow Center, Jerusalem, Israel; c Orthopedic Department, University of Washington Medical Center, Seattle, WA, USA

**Keywords:** Radial nerve, Triceps aponeurosis, Deltoid tuberosity, Posterior approach, Humerus fracture, Anatomical landmarks

## Abstract

**Background:**

The radial nerve, originating from the posterior cord of the brachial plexus, traverses the posterior humerus. Incidences of radial nerve injury have been noted following surgical interventions like fracture fixation and exploration in this area. There's a paucity of literature detailing soft tissue anatomical cues for radial nerve dissection. This study aimed to identify reliable soft tissue and bony landmarks (triceps aponeurosis and deltoid tuberosity) that can be of substantial importance in dissecting the radial nerve and reducing iatrogenic nerve injury utilizing the posterior approach.

**Methods:**

Thirty-two fresh-frozen cadaver specimens underwent dissection using a posterior triceps-splitting approach to expose the radial nerve. The distance between the apex of the triceps aponeurosis and the radial nerve was measured, alongside noting the radial nerve's position relative to the deltoid tuberosity.

**Results:**

Of the cadavers, 78% were female, and 22% were male, with a mean age of 76 (range: 62-85). The average distance between the aponeurosis apex and the radial nerve was 40.3 mm (range: 28-60). The radial nerve was consistently found in all specimens, situated posteriorly at the humerus's mid-axial level at the distal part of the deltoid tuberosity.

**Conclusion:**

The triceps aponeurosis and distal deltoid tuberosity serve as reliable and practical landmarks for dissecting and exploring the radial nerve during posterior humeral approaches. These landmarks prove especially valuable when fractures obscure conventional anatomical cues.

The radial nerve arises from the posterior cord of the brachial plexus, passes posterior to the humeral diaphysis in the spiral groove, pierces the lateral intermuscular septum to step inside the anterior compartment of the arm, and after that, branches near the radio-capitular joint into the posterior interosseus nerve and the radial sensory nerve.^5^

Fractures of the humerus account for 5%-8% of all fractures.^7^ Some features are treated through nonsurgical, while others require surgical intervention.^16^ Identifying and protecting the radial nerve is crucial when surgical treatment is elected. The radial nerve injury is caused by both fractures of the shaft of the humerus and iatrogenic during operative fixation of these fractures.^4^^,^^14^ This is most likely to occur if the fracture is near the junction of the middle and distal thirds of the humerus, as described by Holstein and Lewis.^6^ It's worth mentioning that despite there are various approaches, the posterior approach remains commonly utilized to fix mid and distal humerus fractures with compression or locking plates.^4^ Injuries to the radial nerve were reported at rates ranging from 1.9% to 3.3% following humerus fractures and as high as 11.5% after surgery for humerus nonunion.^8^^,^^9^ As the radial nerve is closely related to the humerus, it leads to Iatrogenic injury related to exposure and fixation devices along the middle third and distal third of the humerus.^18^

Knowledge about the radial nerve anatomy and its relation with various anatomical landmarks helps the surgeon to reduce accidental injury to the nerve or its branches.^15^ Historically, there has been significant interest in using bony landmarks to guide the anatomic localization of the radial nerve. While these landmarks are helpful for preoperative planning and the initial skin incisions, they become less relevant at the deeper dissection layers.^17^ Additionally, surgical draping can make landmarks, like the acromion, challenging to localize intraoperatively.^12^ Furthermore, complex humerus fractures have the potential to alter these measurements, leading to inaccuracies. It has been suggested that alternative soft tissue landmarks may be superior to bony landmarks as they are more likely to remain consistent after trauma.^11^^,^^12^^,^^17^^,^^3^^,^^1^ Various studies described lateral epicondyle, medial epicondyle, and acromion as beneficial anatomical landmarks for dissecting the radial nerve.^1^ A paucity of literature has described the relationship between the deltoid tuberosity, triceps aponeurosis, and the location of the radial nerve.

This study aims to assess the position of the radial nerve to a soft-tissue landmark (triceps aponeurosis) and a bony landmark (deltoid tuberosity) in the arms of fresh cadavers. Blending soft tissue and bony landmarks enhances the surgeon's grasp of the radial nerve's anatomy. This integration aims to mitigate the chances of iatrogenic nerve injury and alleviate the challenges in dissecting the radial nerve.

## Methods

This study included thirty-two adult fresh-frozen upper extremity cadaveric specimens from elderly donors. The upper extremity specimens included the shoulder girdle: the clavicle, the entire scapula, the associated musculature, and the rest of the upper extremity distally.

Arms with deformation, previous surgical incisions, or deformity were excluded from the study. Both female and male and right and left arm cadavers were used. A single orthopedic shoulder and elbow surgeon dissected them using 2.5 magnification loupes. Two surgeons conducted separate measurements simultaneously and subsequently reached a mutual consensus on the measurements. Surgical dissection was performed using a triceps-splitting posterior approach to the humerus in the lateral position (mimicking the surgical posture of the arm in lateral decubitus). The area where the radial nerve passed across the posterior aspect of the humerus was defined as the zone of interest during dissection and data collection. In the zone of interest, the relationship between the radial nerve (mid-axial point), triceps aponeurosis, and deltoid tuberosity was carefully measured and documented using a digital caliper. The proximal and distal part of the deltoid tuberosity was exposed, and the relation with the radial nerve was observed and recorded.

## Results

**Figure 1:**
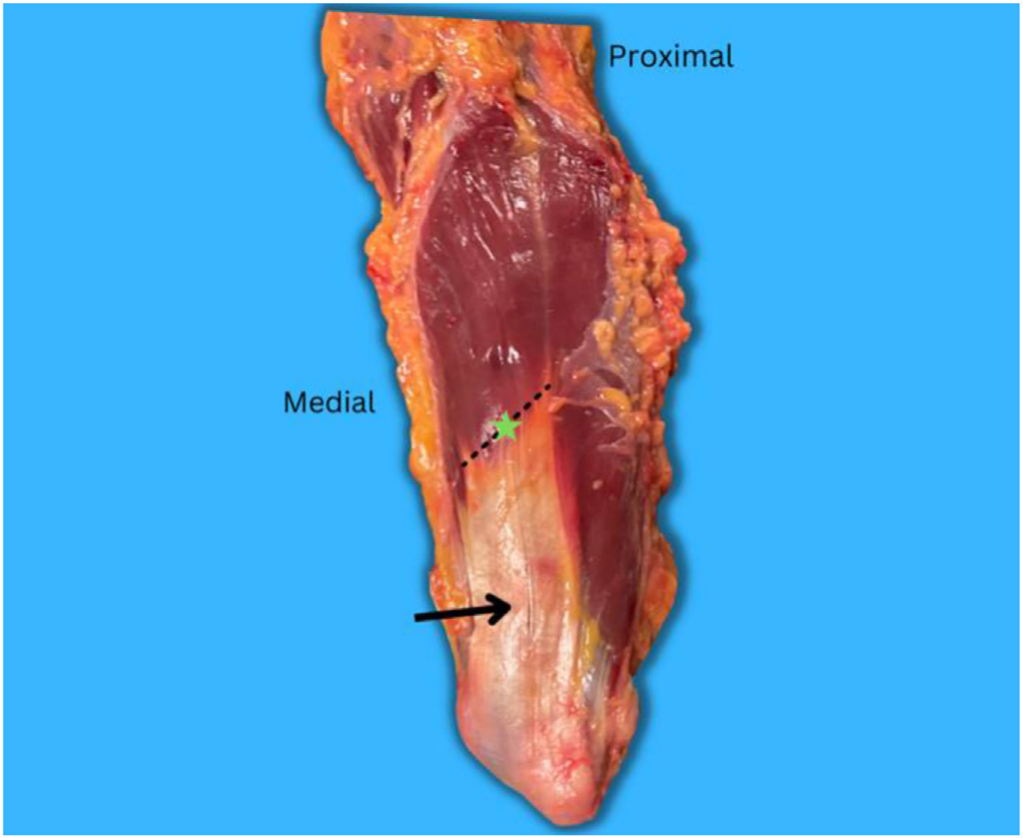
Depicts the triceps aponeurosis anatomy. The () denotes the aponeurosis, while the () connects its medial and lateral edges. The () marks the midpoint of this (), serving as the reference point for measurements.

**Figure 2:**
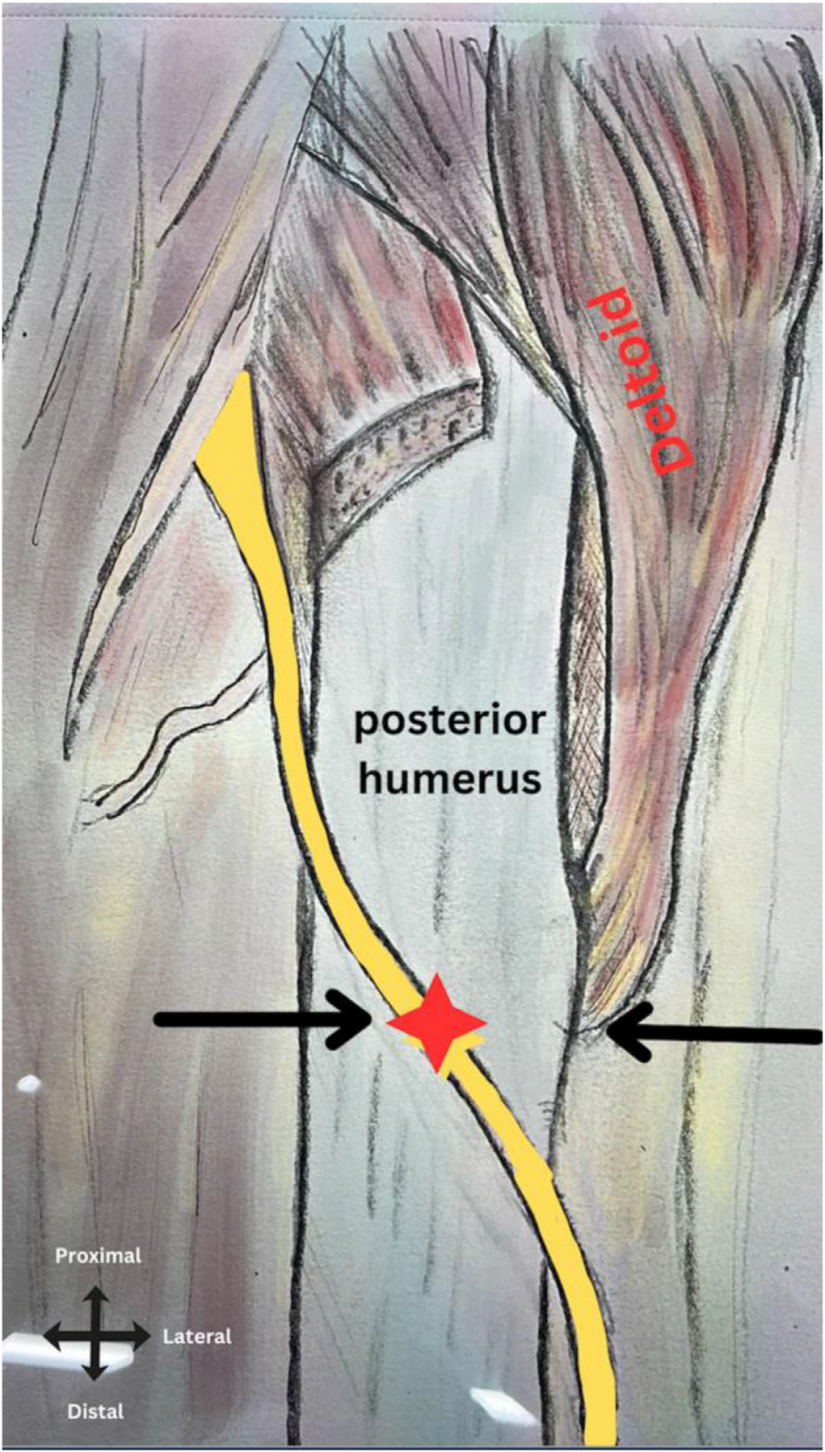
Illustrates the positioning of the radial nerve () in relation to the distal aspect of the deltoid tuberosity ().

The triceps aponeurosis among the cadavers was present in all the specimens (Fig. 1). Thirty-two cadavers were dissected (Table I). 78% of the cadavers were female, and 22% were males. The mean age is 76 years (ranges 62-85). The mean distance of the radial nerve from the aponeurosis was 40.3 mm, with a range of 28-60 mm (Table II). The minimum distance was found to be 28 mm, so this distance was suggested to be the maximum length of the triceps split. There was no significant difference between the right and left hands; the mean was 39.9 mm for the right hands and 40.8 mm for the left (*P* = .42). In all the cadavers (100%), the radial nerve was consistently positioned at the posterior mid-axil point of the humerus, aligned with the distal part of the deltoid tuberosity (Fig. 2).

**Table I:** Data for the first 18 cadavers.

Sample number	Gender	Side	Distance “mm”
1	F	R	30
2	F	R	51
3	F	R	35
4	F	R	35
5	M	R	40
6	M	R	45
7	F	R	35
8	F	R	31
9	F	R	31
10	F	R	60
11	M	R	51
12	F	L	29
13	F	L	30
14	F	L	50
15	F	L	28
16	F	L	40
17	F	L	55
18	M	L	50

*M*, male; *F*, female; *R*, right; *L*, left.

**Table II:** Displays the measurements of the radial nerve concerning the triceps aponeurosis.

Mean	40.3
Range (max-min)	60-28
Median	37.5
SD	10.2
Mean for R only	39.9
Mean for L only	40.8
*P* value (left, right)	.42

*SD*, standard deviation; *R*, right; *L*, left.

**Figure 3:**
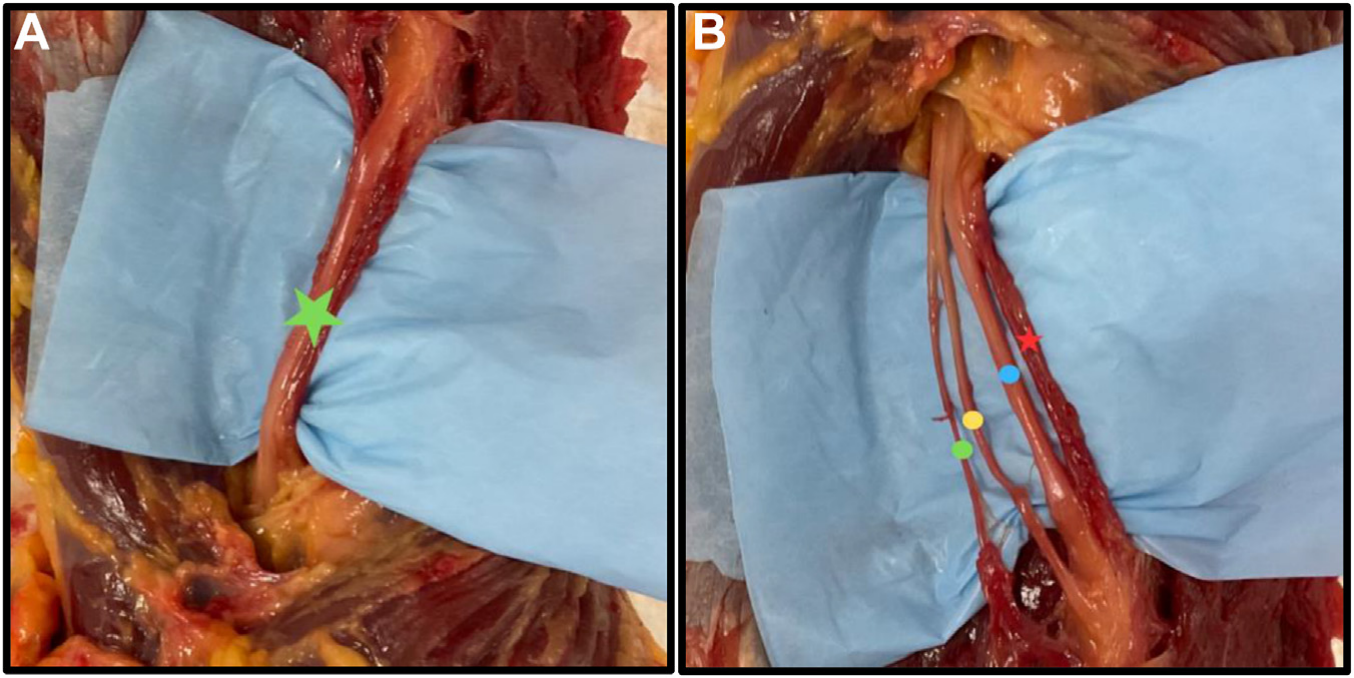
Presents (**A**) the radial nerve proper () enclosed within a fibrous sheath prior to dissection and (**B**) the contents of the sheath postdissection. Notably, the () signifies the vein and artery, the () indicates the radial nerve proper, the () represents the sensory branch (posterior antebrachial cutaneous nerve), and the () denotes the branch for the medial head of the triceps.

In all dissections, the radial nerve proper lays within a fibrous sheath that contains a vein, artery, a sensory branch (posterior antebrachial cutaneous nerve), and a branch for the medial head of the triceps (Fig. 3).

## Discussion

Most studies assessed the relationship between the radial nerve and the bony landmark. However, a paucity of studies evaluated the soft-tissue relation with the radial nerve.^11^^,^^12^^,^^17^^,^^3^^,^^1^ This study aimed to determine the quantitative relationship between the tip of triceps aponeurosis and the radial nerve and define the relationship with the deltoid tuberosity. Thirty-two fresh frozen cadaveric upper extremities were dissected. The radial nerve's mean distance from the aponeurosis tip was 40.3 mm. In addition, the radial nerve was positioned in all the cadavers at the distal part of the deltoid tuberosity at the posterior mid-axial part of the humerus. These findings assist in predicting the location of the radial nerve, potentially reducing the likelihood of iatrogenic injury and streamlining nerve dissection. Furthermore, it guides minimally invasive procedures and the application of external fixators.

There are some difficulties localizing the radial nerve when the classical bony landmark (lateral epicondyle, medial epicondyle, acromion) is disrupted after trauma. Preincision marks often become challenging to identify intraoperatively.^12^ Measurements taken from the medial and lateral epicondyles don't aid surgeons in pinpointing the nerve's location where it crosses the humerus rather than its entry and exit points along the radial groove.

Fractures can disrupt the relationship between bony landmarks and the radial nerve, rendering these relationships unreliable for nerve dissection.^12^^,^^17^ Patra et al have found that the relationship of the radial nerve with osseous landmarks had minimal correlative value, a wide interobserver variability, and was challenging to apply intraoperatively.^11^

**Figure 4:**
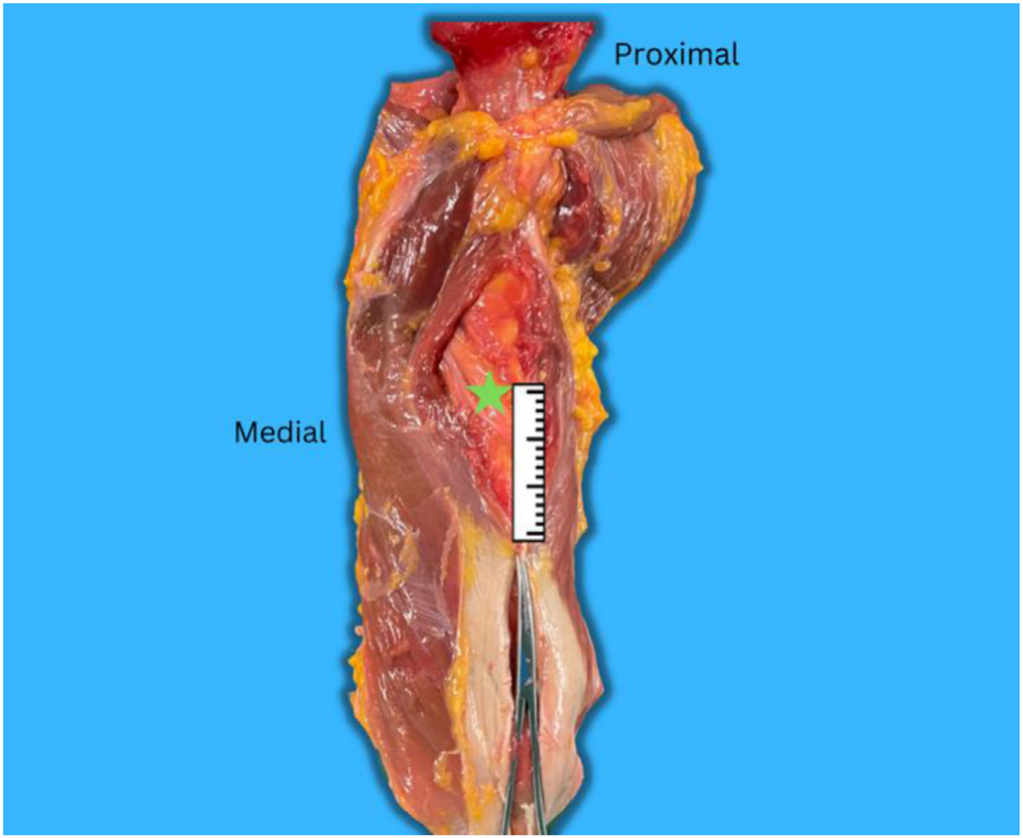
Shows the distance between the radial nerve () and the triceps aponeurosis.

Our study shows that the mean distance from the aponeurosis tip to the radial nerve is 40.3 mm (Fig. 4), with a 28-60 mm range. Similarly, a study by Patra and his colleagues concluded that soft tissue landmark (the distance from the triceps aponeurosis confluence to the radial nerve) is reliable, and the mean is 3.59 ± 0.16 cm^12^. Their study used 20 formalin-embalmed cadavers to measure the distance of the lateral epicondyle-radial nerve, acromion-radial nerve, and triceps aponeurosis-radial nerve; they concluded that soft tissue landmark is the most reliable and accurate to locate the radial nerve. Seigerman et al found similar measurements; the distance was 39.0 mm (range, 36-44 mm), approximating two finger breadths proximal to the tip of the triceps aponeurosis.^17^ Also, in 2018, Prasad et al reported a distance of 39.7 ± 11.8 mm between the confluence point of the triceps aponeurosis and the radial nerve.^13^

Arora and his colleagues documented a shorter mean distance for the radial nerve to the tip of aponeurosis (2.51 cm).^1^ their study included Indian cadavers, and they had a limited number of cadavers (ten), which could explain the difference between their results and our study's findings. In addition, this statement on variation was echoed by McCann et al.^10^ They noted that the triceps aponeurosis of specimens in their research predominantly had two apices, a medial/proximal apex and a lateral/distal apex. When measuring the distance from the radial nerve to the medial apex, the authors reported a value of 21.8 ± 8.2 mm. This value was within the range reported by Arora et al, which is 18.5 mm lower than our study's mean values.^1^ Therefore, McCann et al postulated that the difference in values reported was due to variation in what was defined as the triceps apex or point of confluence.^10^ Our study used the mid-distance between the medial and lateral apex as a reference point, as shown in Figure 1.

While there are various measurements, these studies all suggest that the apex of the triceps aponeurosis has value in localizing the radial nerve during the posterior approach. The radial nerve is about two finger breadths proximal to the tip of the triceps aponeurosis, and distal to the triceps aponeurosis is considered a safe zone.

The relation of the radial nerve to the deltoid tuberosity was assessed to increase the precision of locating the radial nerve. In all the specimens, the radial nerve was found mid-axial of the posterior humerus at the level of the distal part of the deltoid tuberosity. Consistent findings across all the cadavers reaffirm the reliability of the distal deltoid tuberosity as a practical landmark for locating the radial nerve. Similarly, Carlan et al concluded that the distal part of the deltoid tuberosity is a reliable and helpful landmark that can be used to locate the radial nerve.^2^ Thus, minimal posterior cortical penetration is recommended for fracture fixation in the area at the distal deltoid tuberosity to minimize the risk of chronic nerve irritation or iatrogenic injury. The amalgamation of the distance from the aponeurosis tip and the distal deltoid tuberosity presents a practical landmark aiding radial nerve dissection during posterior approaches.

This study has limitations owing to its limited sample size and the variable anatomy present in elderly cadavers, such as muscle bulk and the potential presence of prior shoulder injuries, arthritis, or scar tissue. Moreover, extrapolating specific clinical outcomes from cadaveric findings is challenging. However, the consistency of these results suggests the potential of these anatomical landmarks as guides for surgical approaches. Another limitation of the study is the absence of an interobserver reliability test, with observer consensus being relied upon instead. Further clinical studies are needed to expand on these findings and measure the distance between the deltoid tuberosity and radial nerve.

## Conclusion

This study's findings demonstrate that this soft tissue landmark (mean 40.3 mm proximal to the triceps aponeurosis), coupled with the deltoid tuberosity, aids in predicting the radial nerve's location, potentially reducing iatrogenic injuries and facilitating nerve dissection in surgery.

## Acknowledgment

The authors wish to express our appreciation to Fadi Shweiki, MD, for his assistance in creating the figure.

## Disclaimers:

Funding: No outside funding or grants were used for the completion of this study.

Conflicts of interest: The authors, their immediate families, and any research foundation with which they are affiliated have not received any financial payments or other benefits from any commercial entity related to the subject of this article.
